# A Staging Scheme for the Development of the Scuttle Fly *Megaselia abdita*

**DOI:** 10.1371/journal.pone.0084421

**Published:** 2014-01-07

**Authors:** Karl R. Wotton, Eva Jiménez-Guri, Belén García Matheu, Johannes Jaeger

**Affiliations:** EMBL/CRG Research Unit in Systems Biology, Centre for Genomic Regulation (CRG), Universitat Pompeu Fabra (UPF), Barcelona, Spain; University of Otago, New Zealand

## Abstract

Model organisms, such as *Drosophila melanogaster*, provide powerful experimental tools for the study of development. However, approaches using model systems need to be complemented by comparative studies for us to gain a deeper understanding of the functional properties and evolution of developmental processes. New model organisms need to be established to enable such comparative work. The establishment of new model system requires a detailed description of its life cycle and development. The resulting staging scheme is essential for providing morphological context for molecular studies, and allows us to homologise developmental processes between species. In this paper, we provide a staging scheme and morphological characterisation of the life cycle for an emerging non-drosophilid dipteran model system: the scuttle fly *Megaselia abdita*. We pay particular attention to early embryogenesis (cleavage and blastoderm stages up to gastrulation), the formation and retraction of extraembryonic tissues, and the determination and formation of germ (pole) cells. Despite the large evolutionary distance between the two species (approximately 150 million years), we find that *M. abdita* development is remarkably similar to *D. melanogaster* in terms of developmental landmarks and their relative timing.

## Introduction

Much work in developmental biology has focused on a small number of model organisms, such as the vinegar fly *Drosophila melanogaster* [1]–[3]. While limiting our focus to such models can lead to a more profound molecular understanding of specific embryological processes [3], there are several good reasons to embrace a broader comparative approach including less well-established experimental systems [1]. First and foremost, developmental (and other biological) processes are diverse, and limiting ourselves to the study of model organisms severely restricts our capability to appreciate and study this diversity. Second, without proper evolutionary context, it is impossible to understand the origin and history (and hence the idiosyncrasies) of any developmental process. Only a higher sampling of different species will provide the proper context in which to understand results obtained in classical experimental models. Last but not least, a comparative approach is absolutely essential for understanding the principles underlying the function of regulatory networks responsible for development. The same outcome—e.g. axis determination, segmentation, or organ formation—can be achieved in numerous different ways at the genetic and molecular level. Only by comparing homologous processes in different species will we be able to identify and analyse those aspects of development that have (or do not have) to be conserved to ensure a specific viable phenotype. For these reasons, it is important to develop new species—beyond classical models such as *D. melanogaster*—which are amenable to experimental investigation in the laboratory.

One important step towards establishing a new experimental model system for developmental biology is to provide a careful description and staging scheme for embryogenesis into which molecular experimental findings can be placed. However, this step is often neglected. Many staging schemes in current use are based on the classical embryological literature, but it is difficult to find recent examples of systematic and integrated descriptions of embryological development for any established or emergent model organisms. One example for this trend is the flour beetle *Tribolium castaneum*. Although a staging scheme for a closely related species was published in 1970 [4], and several studies have since contributed to the characterisation and understanding of specific morphogenetic processes (see, for example, [5]–[8]), there is still no detailed, systematic, and integrated description of this model organism's development and life cycle.

In this and the accompanying paper by Jiménez-Guri *et al.* [9], we attempt to counter this trend by providing a detailed developmental schedule, staging scheme, and morphological characterisation of the life cycle of two non-drosophilid dipteran species that we use as experimental models in our laboratory: the scuttle fly *Megaselia abdita* (this paper), and the moth midge *Clogmia albipunctata* [9].

The scuttle fly *Megaselia abdita* belongs to the family Phoridae (hump-backed flies) whose lineage is part of the dipteran sub-order of Brachycera. Phylogenetic analysis has identified the phorids as belonging to the earliest branching lineage in the radiation of the cyclorrhaphan flies, forming part of the paraphyletic assemblage of Aschiza [10], [11]. They diverged from the lineage leading to *D. melanogaster* approximately 150 million years ago [10]. The phorid family is extremely diverse and rich in species, over 4,000 of which have been described so far. Marshall [12] states that “the family Phoridae is like a biodiversity iceberg” referring to the potentially vast number of unnamed and unstudied species. The genus Megaselia forms one of the largest groups among the phorids. Its distribution is cosmopolitan. *M. abdita* is often found along with another Megaseila species, the coffin fly *M. scalaris*, feeding on carrion—including human bodies—which has led to its widespread use in forensics [13]–[16]. Due to their largely subterranean lifestyle, both species are better runners than fliers. Despite their jerky scuttling movement and hump-backed appearance, the Megaselia genus was described as “[h]ow a fly ought to be” by Richard Dawkins in his book “The Ancestor's Tale” [17].

Over the past few years, *M. abdita* has been gaining popularity as a model species in the field of evolutionary developmental biology. In particular, it has been used to investigate the evolution of antero-posterior axis patterning [18], segment determination [19]–[21], head patterning [22], mRNA localisation [20], BMP signaling [23], [24] and the formation and morphology of extraembryonic tissues [24]–[26]. Recently, high-throughput sequencing data has also become available from a transcriptomic analysis of early embryos [11], while efforts to sequence the *M. abdita* genome are also underway (our unpublished data). Despite this, a systematic characterisation and analysis of its development has yet to be carried out, and a rigorous staging scheme—long available for *D. melanogaster* (reviewed in [27])—has been lacking.

In this paper, we present an overview of the *M. abdita* life cycle, as well as a detailed description of its embryonic development with a special focus on early embryogenesis (cleavage and blastoderm stages up to gastrulation), the formation of extraembryonic tissues, and the determination and formation of germ (pole) cells. Wherever possible, we homologise processes to the established *D. melanogaster* staging system.

## Results and Discussion

We characterised the development of *M. abdita* through observation and timing of life stages using live imaging microscopy. Selected stages were examined in more detail by imaging stained fixed embryo samples and by scanning electron microscopy.

### The life cycle of *M. abdita*

**Figure 1:**
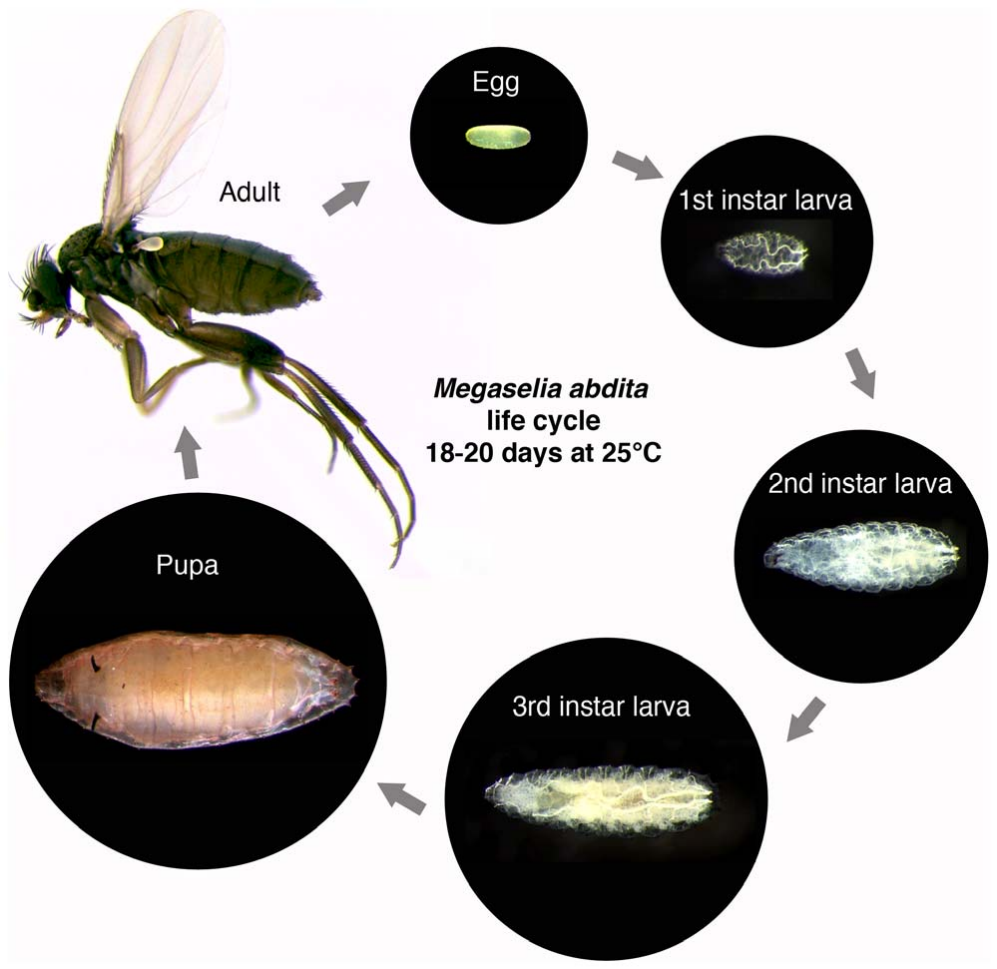
The life cycle of *M. abdita*. Embryonic development is covered in detail in the main text of the paper. After hatching, *M. abdita* goes through three larval instars before forming a pupa. The whole life cycle takes 18–20 days to complete.

An outline of the *M. abdita* life cycle (Figure 1) has previously been described in Rafiqi *et al.* [28]: rearing the animals at 25°C, under a 16/8 hrs day/night cycle and 75% relative humidity, resulted in a life cycle duration of 18–20 days; approximately 24 hrs of this are taken up by embryogenesis, four days by larval development, and 10 days spent in the pupal stage [28]. Under identical conditions, we observed completion of embryogenesis in approximately 24 hours, 5 days for larval development, and 12 days in the pupal stage. Although cuticle moulting was difficult to observe directly, we detected three larval instars. Four days before emerging, the pupa blackened. Adults survived for between 6–10 days. The average time for completion of the life cycle is in the range of around 18 days (*n* = 4).

### Embryonic development: an overview

We used live imaging with differential interference contrast (DIC) to produce a series of movies covering all stages of embryonic development (for examples, see Movie S1 and Movie S2). Microscopy was carried out at 25°C under voltalef oil. Dechorionation of the embryo was necessary to obtain clear DIC images. Under these conditions, embryogenesis lasts approximately 27.5 hrs from oviposition until hatching.

**Figure 2:**
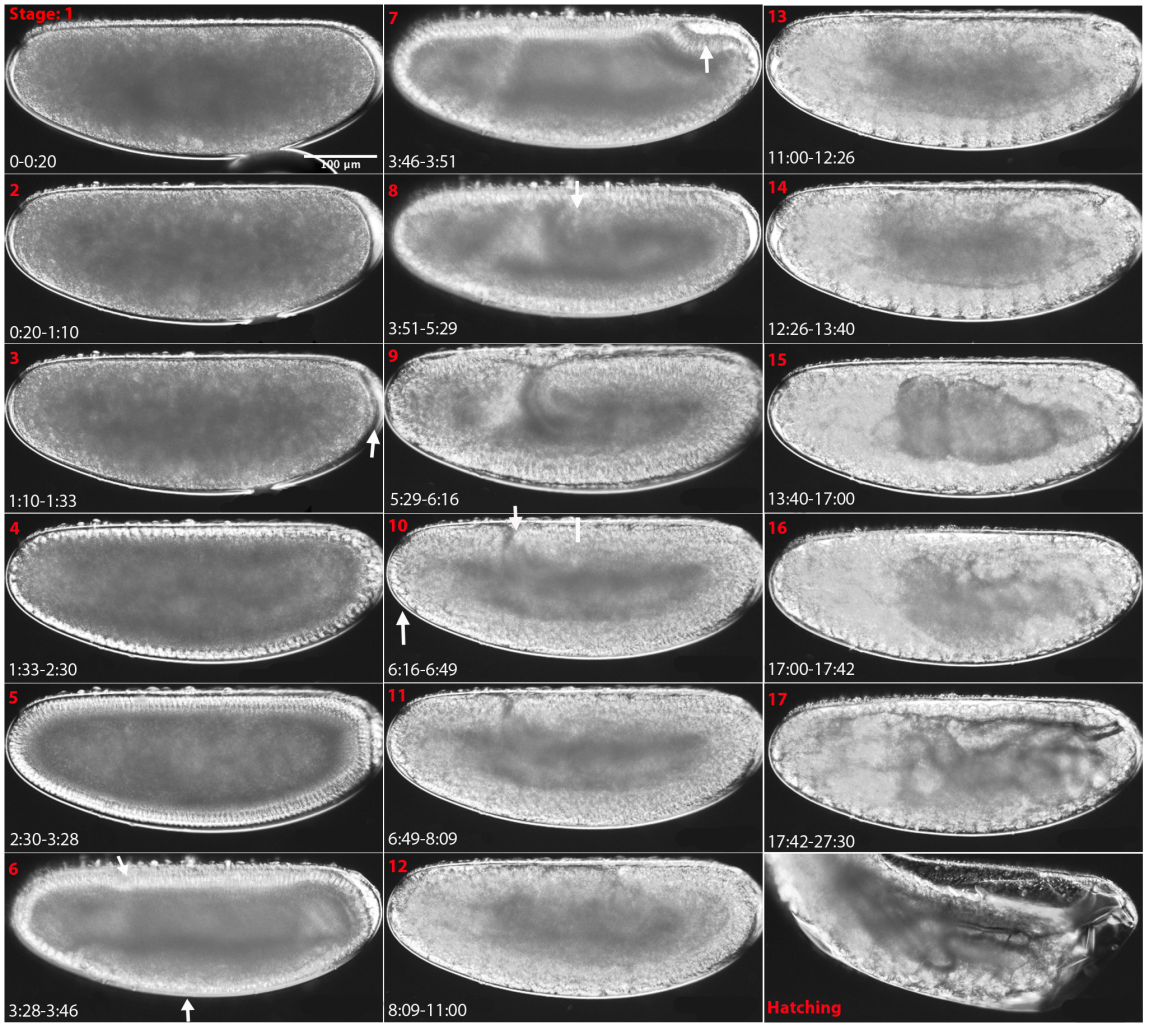
Embryonic staging and developmental events in *M. abdita*. Embryos are shown as lateral views: anterior is to the left, dorsal is up. Stage numbers (roughly corresponding to Bownes' stages in *D. melanogaster* [27]) are shown in red at the top left, and time after egg laying (AEL) in hrs:min in white at the bottom left corner of each panel. White arrows and bars indicate morphological landmarks. See main text for a detailed description, and Figure 3 for comparative timing of stages with reference to *D. melanogaster*.

**Figure 3:**
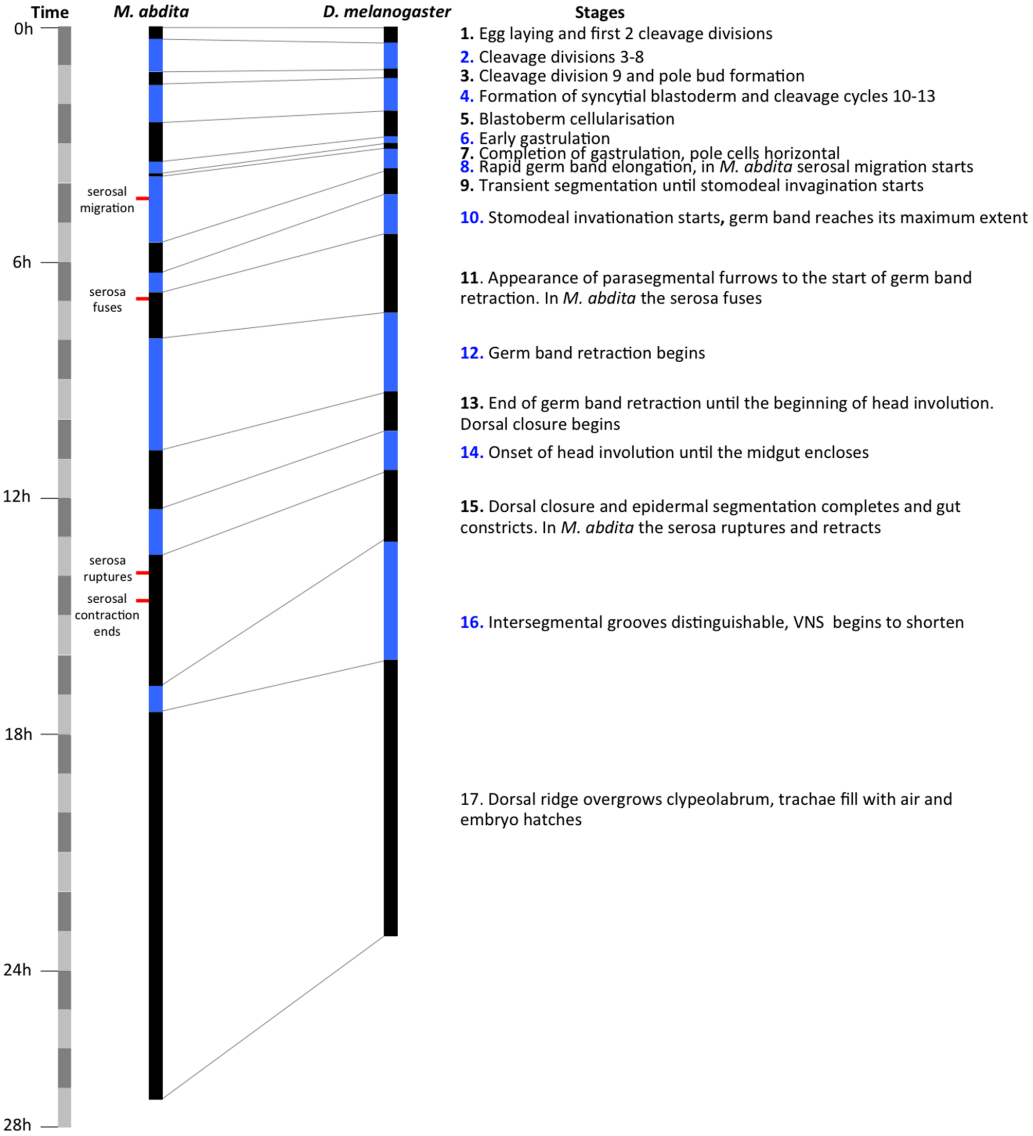
Comparative timing of developmental stages in *M. abdita* and *D. melanogaster*. The duration of each stage is shown for *M. abdita* and *D. melanogaster* in alternating black and blue bars. The time scale is divided into blocks of 1 hr on the far left hand side. A brief description of each stage is given on the right. Landmarks of extraembryonic tissue formation and retractions are indicated to the left of the *M. abdita* time scale.

Development can be divided into 17 stages roughly corresponding to Bownes' stages in *D. melanogaster* [27]. Each stage can be distinguished by distinct morphological markers, as shown in Figure 2 (also see Movie S1). The similarity between *D. melanogaster* and *M. abdita* development allows a direct comparison between developmental stages, as discussed below and shown in Figure 3.

Here we provide an overview over all stages of development. Early development (cleavage and blastoderm stages 1–6 up to gastrulation), extraembryonic tissue formation and retraction (stages 8–15), and pole cell formation (at stage 3), are described in more detail in separate sections below. All times are displayed as hrs:min unless otherwise indicated. To assist identification of stages under different growth conditions (e.g. oil/dry) and at different temperatures, we also supply timing as percentage of total embryonic development (% TED). Raw data for each event including the number of embryos examined (*n*) and standard deviations (SDs) are supplied in File S1. In general, SDs are below 5 min before serosal migration begins during stage 8. After this, there is a trend towards increasing SDs over time: 16 min for the start of serosal migration, 23 min for the start of dorsal closure, 41 min for serosal rupture, 58 min for the start of stage 16, 1:42 for the start of stage 17 and 3:26 for hatching.

#### Stage 1

0:00–0:20 (duration: 0:20, 1.2% TED). Newly laid eggs are approximately 536±31 µm long and 223±22 µm wide (*n* = 164, measured with FlyGUI [29]). This stage begins at egg laying, and lasts until the end of the first two cleavage divisions, at the beginning of cleavage cycle 3 (C3). Experimental constraints—including the time required for egg laying, dechorionation, and mounting—restricted our earliest observation point to partway through C2. Since all cleavage cycles up to C12 are of a very similar duration (approximately 10 min), we infer stage 1 to last for at least 20 min. All ‘times after egg laying (AEL)’ below include a correction based on this estimate (see File S1 for raw timing data, and exact time adjustment values). In *D. melanogaster*, stage 1 occurs over a 25 min period (1.4% TED; see [27] for references to Bownes' stages during *D. melanogaster* development).

#### Stage 2

0:20–1:10 (duration: 0:50, 3% TED). Cleavage cycles C3 to C8 take place. During this time, an empty space appears between the vitelline membrane and the egg cytoplasm at the posterior pole. We were unable to accurately time the retraction from the posterior pole but observe its disappearance along with the formation of the pole cells at stage 3 (see below). In *D. melanogaster*, stage 2 occurs from 0:25–1:05 and takes 0:40 (3% TED) during which egg cytoplasm can be seen retracting from the vitelline envelope at both poles; filling of the space at the posterior pole occurs at stage 3.

#### Stage 3

1:10–1:33 (duration: 0:23, 1.4% TED). Stage 3 includes cleavage cycle C9 and the beginning of C10. At this stage, nuclei divide and migrate outwards, and the pole buds form (Figure 2, stage 3, white arrow). Stage 3 ends with the arrival of nuclei at the periphery of the embryo. During this time, an empty space appears between the vitelline envelope and the egg cytoplasm at the anterior pole at around 1:13 and persists until stage 4 (1:38). In *D. melanogaster*, this stage occurs from 1:05–1:20 and lasts for 0:15 (1% TED). During this stage, the empty space at the posterior of the embryo disappears in both species.

#### Stage 4

1:33–2:30 (duration: 0:57, 3.4% TED). At the onset of this stage, the nuclei have reached the periphery and form the syncytial blastoderm. Metaphase (or pseudo-cleavage) furrows form around each nuclei before the breakdown of the nuclear envelope during cleavage cycles C10–13 (see also below). Stage 4 terminates at the beginning of cleavage cycle C14. In *D. melanogaster*, the syncytial blastoderm stage occurs from 1:20–2:10 and lasts for 0:50 (3.5% TED).

#### Stage 5

2:30–3:28 (duration: 0:58, 4% TED). Similar to previous blastoderm cycles, cellular membranes begin to form at cleavage cycle C14, and progressively grow to engulf the elongating blastoderm nuclei forming the cellular blastoderm. Nuclear morphology changes from circular to elongated (see below). Stage 5 ends just before the onset of gastrulation, and is marked by the wavy appearance of the ventral blastoderm cells (seen as uneven apical and basal surfaces), and the slight dorsal movement of the pole cells. In *D. melanogaster*, this stage occurs from 2:10–2:50 and lasts for 0:40 (3% TED).

#### Stage 6

3:28–3:46 (duration: 0:18, 1% TED). Early gastrulation events occur: the ventral and cephalic furrows form (Figure 2, stage 6, white arrows), and the pole cells continue to shift dorsally. Stage 6 ends when the cell plate carrying the pole cells reaches a horizontal position (parallel to the A–P axis). In *D. melanogaster*, this stage occurs from 2:50–3:00 and lasts for 0:10 (1% TED).

#### Stage 7

3:46–3:51 (duration: 0:05, 0.3% TED). This stage begins with the pole cell plate in a horizontal position. The plate continues to tilt, forming a pocket (the amnioproctodeal invagination; Figure 2, stage 7, white arrow). The beginning of cephalad (headwards) movement of this invagination marks the end of stage 7. The dorsal folds and amnioproctodeal invagination are less conspicuous in our *M. abdita* movies than in *D. melanogaster* although the posterior transverse furrow is clearly visible in fixed embryos counterstained with DAPI (see also below). In *D. melanogaster*, this stage occurs from 3:00–3:10 and lasts for 0:10 (1% TED).

#### Stage 8

3:51–5:29 (duration: 1:38, 6% TED). This stage starts with the cephalad movement of the amnioproctodeal invagination, marking the onset of the rapid phase of germband extension. The germband reaches approximately 50% A–P position (Figure 2, stage 8, white arrow; 0% A–P position is at the anterior pole), and the amnioserosal lip forms (also see below). Originating from this lip, the serosa migrates to eventually engulf the entire embryo (at stage 11). Stage 8 ends with the transient appearance of mesodermal segmentation. In *D. melanogaster*, this stage occurs from 3:10–3:40 and lasts for 0:30 (2% TED). During this time, the germband reaches beyond 40% A–P position. On the other hand, no serosal migration occurs, since extraembryonic tissues are reduced to a dorsal amnioserosa in *D. melanogaster*, which does not evaginate or migrate.

#### Stage 9

5:29–6:16 (duration: 0:47, 3% TED). The germband continues to extend albeit at a slower rate (slow phase of germband extension), and the serosa continues to migrate ventrally. Stage 9 ends with the formation of the stomodeal invagination (seen more clearly in Figure 2, stage 10, ventral-anterior white arrow). In *D. melanogaster*, this stage occurs from 3:40–4:20 and lasts for 0:40 (3% TED).

#### Stage 10

6:16–6:49 (duration: 0:33, 2% TED) During this stage, the stomodeum continues to form and the germband reaches its maximum extent, around 30% A–P position (Figure 2, stage 10, dorsal white arrow; compare to germband position at stage 8, indicated by a white bar). Stage 10 ends with the appearance of parasegmental furrows. During this time, the serosa fuses ventrally at a posterior position (6:51). In *D. melanogaster*, this stage occurs from 4:20–5:20 and lasts for 1:00 (4% TED), during which the germband reaches its maximum extent at 25% A–P position.

#### Stage 11

6:49–8:09 (duration: 1:13, 4% TED). Stage 11 begins with the appearance of parasegmental furrows, and ends with the beginning of germband retraction. During this time the serosa fuses forming a complete extraembryonic layer around the embryo. The serosa remains intact for around 7 hrs until finally breaking during stage 15. In *D. melanogaster*, this stage occurs from 5:20–7:20 and lasts for 2:00 (8% TED).

#### Stage 12

8:09–11:00 (duration: 2:51, 10% TED). During this stage, the germband retracts. Stage 12 ends with the completion of this process. In *D. melanogaster*, this stage occurs from 7:20–9:20 and lasts for 2:00 (8% TED).

#### Stage 13

11:00–12:26 (duration: 1:26, 5.2% TED). Stage 13 lasts from the completion of germband retraction until the onset of head involution. During this time, the dorsal opening of the embryo remains covered by the amnion, and the serosa envelopes the entire embryo. Dorsal closure starts at the same time as the lengthening of the gut. In *D. melanogaster*, this stage occurs from 9:20–10:20 and lasts for 1:00 (4% TED), during which the dorsal egg surface remains open and the dorsal hole is covered by the amnioserosa.

#### Stage 14

12:29–13:40 (duration: 1:14, 4.5% TED). Stage 14 starts at the beginning of head involution, and ends with closure of the midgut. The head continues to involute beyond the end of this stage, and this process completes only by the time the serosa ruptures at stage 15. In *D. melanogaster*, stage 14 occurs from 10:20–11:20 and lasts for 1:00 (4% TED).

#### Stage 15

13:40–17:00 (duration: 3:20, 12% TED). Stage 15 starts at the closure of the midgut, and covers the completion of dorsal closure and dorsal epidermal segmentation. This stage ends when intersegmental grooves can be distinguished at mid-dorsal levels. During this time, the serosa ruptures at a ventro-posterior position (13:49). During its retraction, the serosa first rounds the posterior pole before rounding the anterior pole, to be contracted into the dorsal hole 40 min after rupturing (see also below). Dorsal closure completes and dorsal epidermal segmentation is visible. Also during this stage, the ventral nerve cord (VNC) starts to shorten, the gut constricts, and muscle contractions begin. In *D. melanogaster*, this stage occurs from 11:20–13:00 and lasts for 1:40 (7% TED), but shortening of the VNC does not begin until stage 16.

#### Stage 16

17:00–17:42 (duration: 0:42, 3% TED). Stage 16 begins with the appearance of the lateral intersegmental grooves, and ends then the dorsal ridge has completely overgrown the tip of the clypeolabrum (completion of head involution). The VNC continues to shorten; completion of this movement is not clearly detectable and probably continues into stage 17. In *D. melanogaster*, this stage occurs from 13:00–16:00 and lasts for 3:00 (13% TED).

#### Stage 17

18:23–30:02 (duration: 9:54, 36% TED). During this stage, the retraction of the VNC is likely to continue and reach completion. The trachea fill with air at around 22 hrs AEL. The first instar larva hatches at around 27.5 hrs AEL. In *D. melanogaster*, this stage occurs from 16:00–24:00 and lasts for 8:00 hrs (33% TED).

### Detailed staging of early embryogenesis in *M. abdita*

#### *M. abdita* has 14 cleavage cycles

As is the case for *D. melanogaster*, a lot of research on *M. abdita* has focused on the earliest stages of embryogenesis [18]–[22]. For this reason, we have examined the two initial phases of development in more detail: the cleavage and the blastoderm stage, both occurring before the onset of gastrulation. Live imaging enables us to count cleavage divisions backwards from gastrulation. Each division can be detected by the disappearance of nuclear envelopes, and their subsequent reappearance at the beginning of the interphase of each cycle. Our movies only capture 12 cleavage divisions (see Movie S1 and Movie S2). However, it is known that 13 nuclear divisions occur before gastrulation in *D. melanogaster* [27], [30]. This suggests that we may be missing the first cleavage cycle from our analysis, due to the delay caused by preparing and mounting embryos for live imaging (see above).

**Figure 4:**
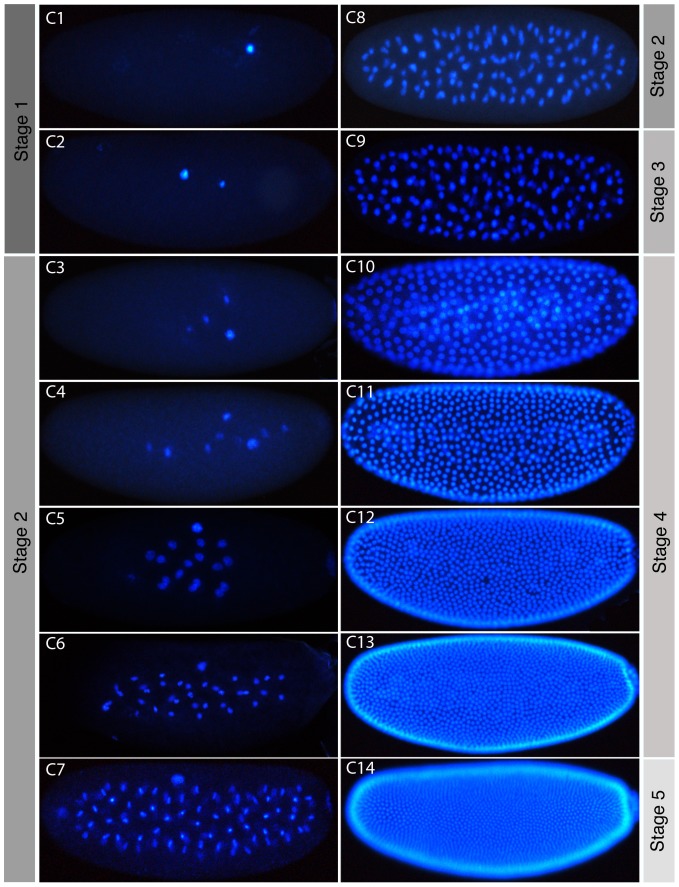
Cleavage cycles of *M. abdita*. Fluorescence images of embryos with DAPI-counterstained nuclei are shown as lateral views. Anterior is to the left. C1–14 indicates cleavage cycle number. The focus is on the sagittal plane for embryos at cleavage stage (C1–C9), and at the surface of the embryo at blastoderm stage (C10–14). As in *D. melanogaster*, nuclei begin to move towards the periphery from C7 onwards. Corresponding embryonic stages (see Figures 2 and 3) are indicated on grey background.

To investigate this—and to confirm our results using an independent approach—we counterstained the nuclei of formaldehyde- or heat-fixed embryos using DAPI (Figure 4). We then imaged and counted nuclei number, comparing our counts to the number expected from the division of a single starting nucleus (Table 1 and File S2). We can clearly identify embryos with a number of nuclei similar to the expected value up to cleavage cycle C8 (2^n^, where *n* is the number of preceding cleavage divisions). After this stage, nuclei begin to reach the periphery, and can no longer be captured in the same plane of focus. Despite this, it is still possible to group embryos into discrete classes with characteristic nuclear numbers representing different cleavage cycles. As nuclei arrive at the surface of the embryo (the yolk-free periplasm) at the beginning of the blastoderm stage, approximately half the expected number is visible in lateral views with a superficial plane of focus (in C10 and C11). This ratio decreases further in C12, which is probably due to our counting method as nuclei increasingly overlap around the periphery of the embryo. Embryos at C12, C13, and C14 can still be clearly distinguished but it becomes somewhat challenging to establish the precise number of nuclei due to dense nuclear packaging. To check our ability to distinguish these embryos we quantified the nuclear density in these embryos. We scaled each embryo to 600×25 mm and counted the number of full nuclei falling into a 45×4 mm square placed in the middle of the embryo. Our counts clearly show nuclear density increasing with each cycle (see density counts in Table 1) and confirm our ability to distinguish between these stages by eye. Taken together, our evidence indicates that *M. abdita* has 13 cleavage divisions, and thus 14 cleavage cycles, just like *D. melanogaster*.

**Table 1:** Observed number of nuclei in *M. abdita*.

Cleavage Cycle	Expected # of Nuclei	*M. abdita* Nucleus Count
C1	1	1±0 (n = 9)
C2	2	2±0 (n = 11)
C3	4	4±0.3 (n = 19)
C4	8	8±0.5 (n = 8)
C5	16	16±1.6 (n = 12)
C6	32	32±3 (n = 8)
C7	64	68±18 (n = 5)
C8	128	110±8.2 (n = 9)
C9	256 (nuclei almost at periphery)	165±41 (n = 7)
C10	512	263±41 (n = 6)
C11	1024	480±47 (n = 5)
C12	2048	∼750 (n = 1) Density: 13 nuclei ±1.8 (n = 7)
C13	4096	∼1200 (n = 1) Density: 22 nuclei ±2.1 (n = 8)
C14	8192	>1400 (n = 1) Density: 32 nuclei ±1.8 (n = 7)

Number counts based on DAPI-stained embryos (Figure 4 and File S2) are compared to those expected considering preceding mitotic divisions. Note that expected numbers are overestimates from C9 onward, as nuclei migrate out of the plane of focus and some remain behind in the yolk (also outside the focal plane). C14 embryos have nuclei, which are too densely packed to be reliably counted. Density refers to the number of full nuclei falling into a 45×45 mm square placed in the middle of a scaled embryo (see text for details).

#### Length and subdivision of blastoderm cycles

Our work on the quantification and mathematical modelling of segmentation gene expression (see, for example, [31]–[41]) requires careful measurements of blastoderm cycle length and a more fine-grained subdivision of cleavage cycle C14A (the portion of C14 before the onset of gastrulation), which lasts significantly longer than the preceding cycles. Previous work in D. melanogaster divided C14A into 8 time classes [38], [39]. Here we choose an analogous approach for M. abdita in order to be able to homologise time classes between species.

**Figure 5:**
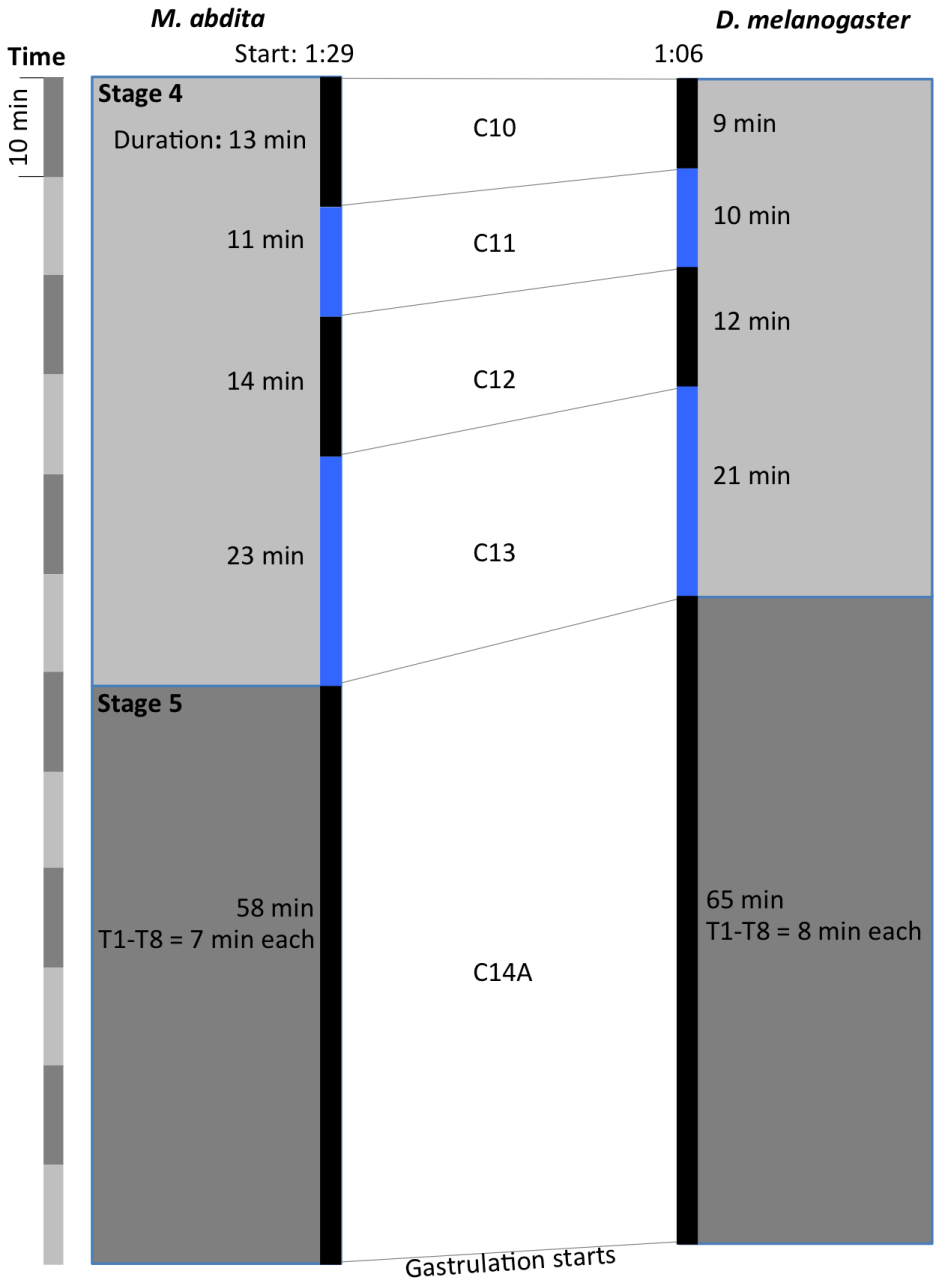
Comparison of the length of blastoderm cycles in *D. melanogaster* and *M. abdita*. The duration of each division cycle is shown for both species with alternating black and blue bars. The onset of each cycle corresponds to the reappearance of nuclear envelopes in DIC movies. The time scale on the left is divided into blocks of 10(in hrs:min after egg laying, AEL) along with duration (in min) are shown for cleavage cycles C10–14. For *D. melanogaster*, time for the start of C10 is taken from [24], and times for the duration of the blastoderm cycles from [27]. Corresponding embryonic stages (see Figures 2 and 3) are indicated on grey background.

We measured the timing and duration of blastoderm cycles C10–14A using DIC live imaging as described above (see Movie S2). The resulting time line is displayed in Figure 5. In *D. melanogaster*, C10 to C14 last for 9, 10, 12, 21, and 65 min respectively [30], [42]. In *M. abdita* the corresponding times are 13, 11, 14, 23, and 58 min.

In addition to measuring cleavage cycle timing and duration, we characterised membrane morphology and nuclear shape in images captured from DIC movies. Single time points during interphase were chosen for cycles C10–13. For the subdivision of C14A into time classes T1–8, we used images captured at eight evenly spaced time points during that cycle.

**Figure 6:**
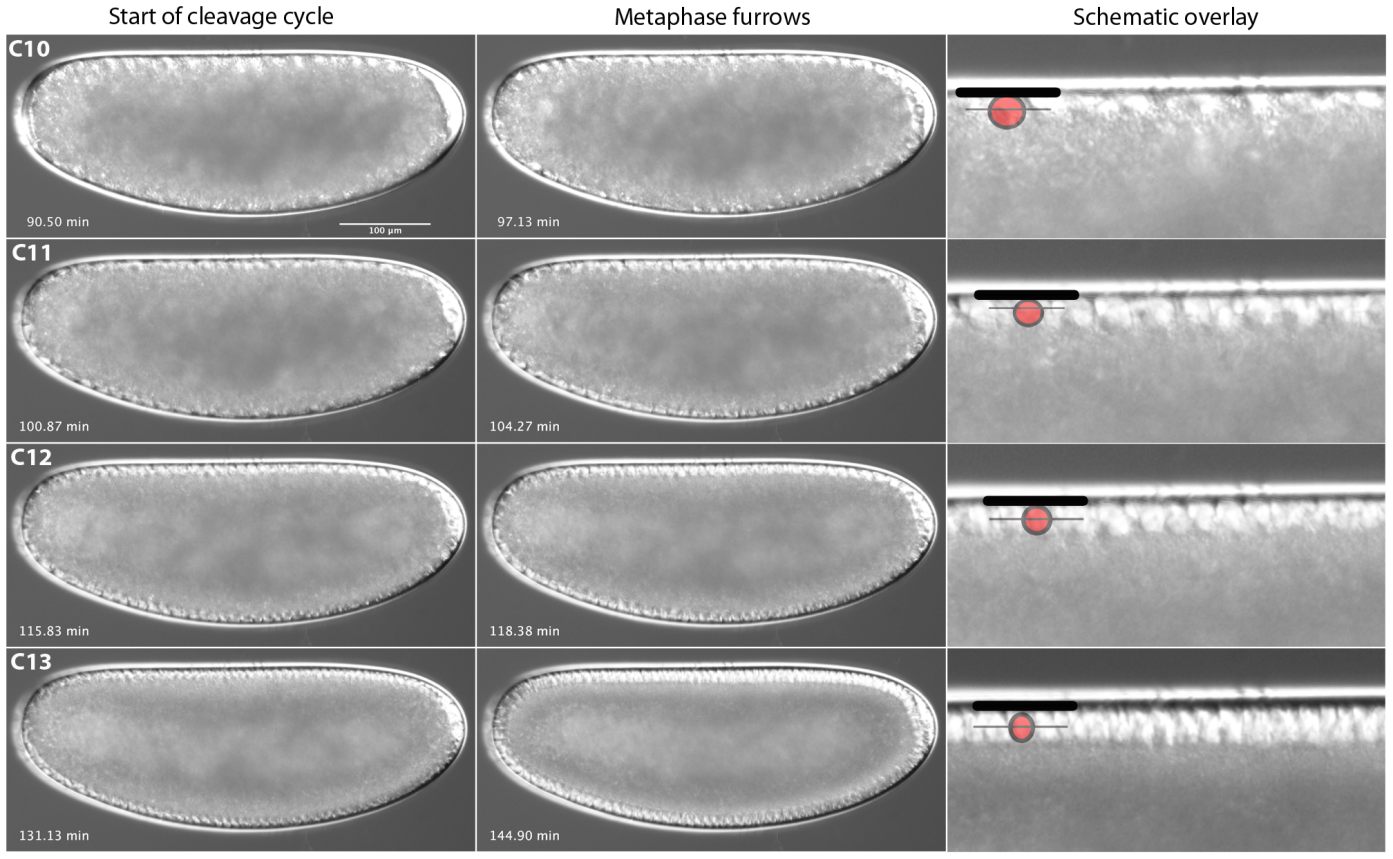
*M. abdita* early blastoderm cycles (C10–13). Captured images from live DIC movies. Images show lateral views, anterior is to the left, dorsal is up. Times in min after egg laying (AEL). Schematic overlays show vitelline membrane (thick black line), nuclei (red circle) and metaphase (pseudo-cleavage) furrow front (thin black line).

**Figure 7:**
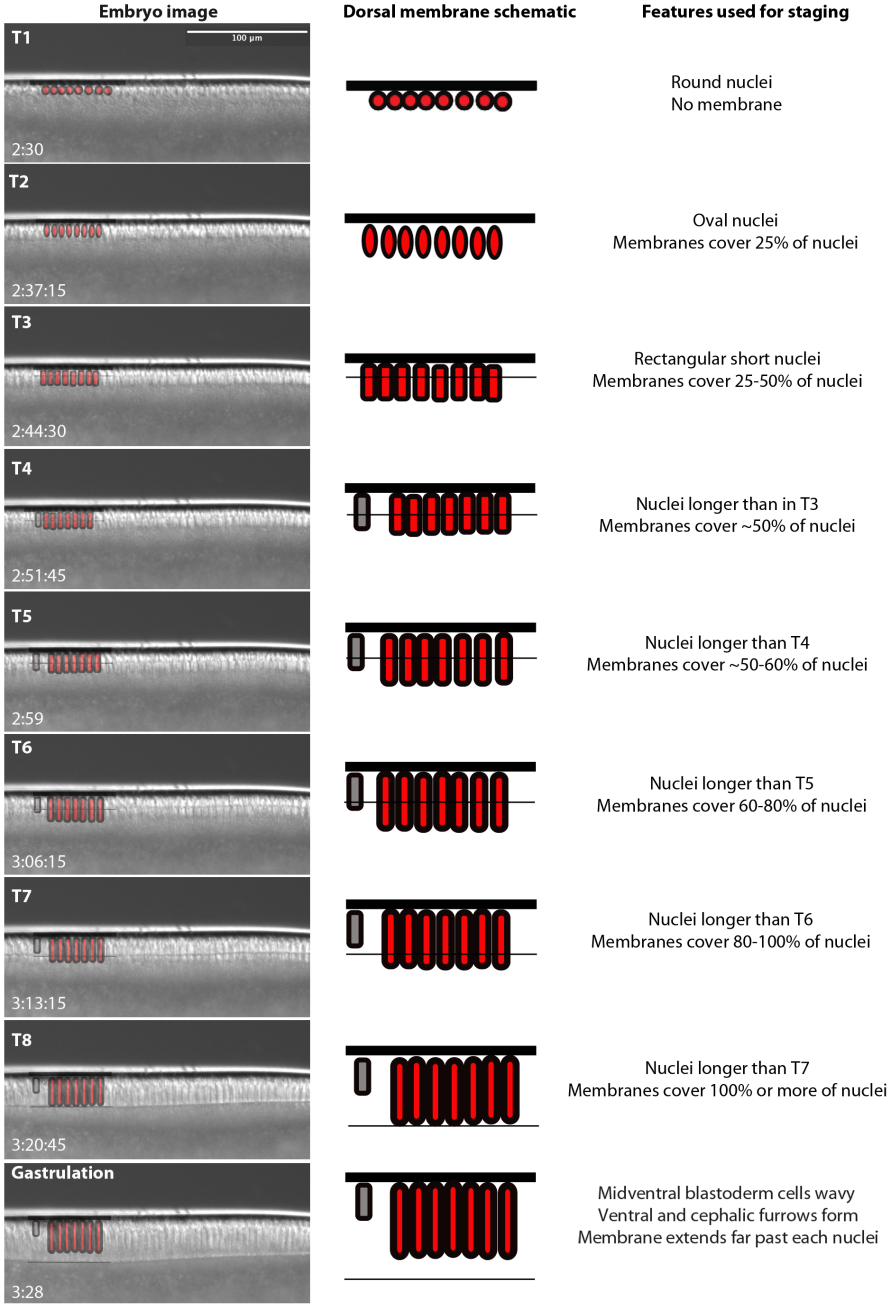
Cellularisation and time classification scheme for *M. abdita* during cleavage cycle 14A. Images captured from time-lapse movies showing the membrane morphology at mid-dorsal positions are shown on the left-hand side for time classes T1–T8 and for gastrulation. Starting times after egg laying for each time class are shown in the bottom left of each image in hrs:min(:sec). Schematic overlays show vitelline membrane (thick black line), nuclei (red circle, oval or rectangle), and invaginating membrane front (thin black line). Grey nuclei indicate the size of the nuclei at T3 for reference. Descriptions of features used to distinguish each stage are provided on the right (see text for details).

From C10 to C13, nucleus number and density in the periplasm increases, but nuclear shape remains in approximately the same rounded state (Figure 6). Metaphase (or pseudo-cleavage) furrows can be distinguished during each of these cycles as in *D. melanogaster* (Figure 6; see also [43], [44]). During cleavage cycle C14A, nuclei change shape and a definitive wave of membrane invagination progresses as cellularisation occurs (Figure 7). At time class 1 (T1), nuclei are still round (as in previous cycles) and no membrane is visible. By T2, nuclei have become oval-shaped and the front of the invaginating membranes has already extended to cover approximately 25% of each nucleus' length. At T3, the nuclei have obtained a short, almost rectangular shape that continues to elongate during the subsequent time classes (compare the original size displayed as a grey reference nucleus with each of the subsequent stages in the schematic drawings in Figure 7). Invaginating membranes cover 25–50% of nuclear length at T3, approximately 50% at T4, 50–60% at T5, 60–80% at T6, 80–100% at T7, and 100% or more at T8. T8 ends with the onset of gastrulation.

#### Staging gene expression*: even-skipped* in the blastoderm

To illustrate the utility of our staging system, we stained and carried out a detailed analysis of the blastoderm-stage expression of the M. abdita pair-rule gene even-skipped (eve) at high temporal resolution. In the D. melanogaster blastoderm, eve shows a very dynamic expression pattern, and can itself be used as marker for the precise staging of embryos [37], [38], [40], [41].

**Figure 8:**
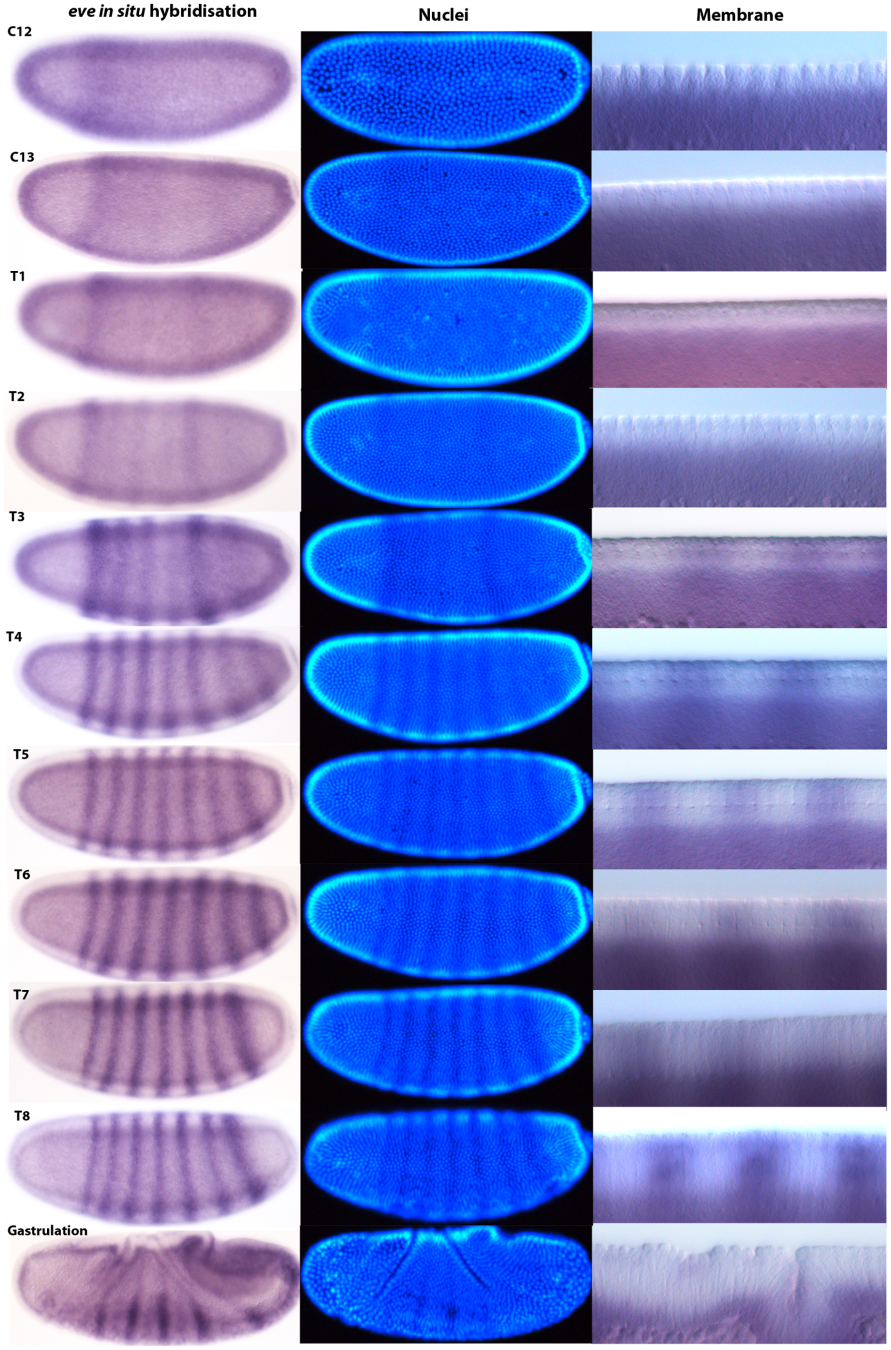
*M. abdita eve* mRNA expression staged using nuclei number, nuclear density, and membrane morphology. Our staging method first distinguishes cleavage cycles based on the number or density of nuclei observed. Dorsal membrane morphology is then used to check the assignment of embryos to cleavage cycles C10–14 based on the size and spacing of the nuclei (see Figure 6). Embryos assigned to cleavage cycle C14A are further classified into time classes T1–8 based on membrane morphology (see Figure 7). Using this method, we provide a detailed time-series for expression of the pair-rule gene *even-skipped* (*eve*) during the blastoderm stage. Lateral views are shown: enzymatic *in situ* hybridisation stains to the left, and DAPI-counterstain in the middle. The right-hand column shows details of dorsal membrane/nuclear morphology (sagittal views). See text for details.

For each time class between C12 and C14A-T8 in *M. abdita*, we captured brightfield and DIC images of whole embryos stained by *in situ* hybridisation against *eve* mRNA (Figure 8), a fluorescent image of the DAPI counterstain, and a higher-magnification DIC image showing details of dorsal membrane morphology (as described in [29]. Time classification was carried out according to nuclear count, shape, and membrane morphology as described in the previous section.

We first detect *M. abdita eve* expression during C12 in a broad expression domain showing relatively strong expression from 25% to 50%, and weaker expression as far back as 80% A–P position. At C13, expression has increased in the posterior (60 to 90% A–P position), and has intensified into a stripe-like domain from approximately 25 to 35% A–P position. At T1 and T2, *eve* stripes 1 and 5 intensify. During T2, weak expression of stripes 2–4 becomes detectable, together with a broad diffuse posterior domain past stripe 5 at 70 to 85% A–P position. Stripe 2 remains joined to stripe 1, and full separation of these stripes only occurs at T4. At T3, stripe 2 and 3 gain in intensity, while stripe 4 and the broader posterior domain remain relatively weaker. A broad domain covering stripes 6 and 7 can be discerned in the posterior region. By T4, stripe 4 has increased its intensity, while the posterior domain has begun to resolve into stripes 6 and 7. By T5, all seven *eve* stripes have formed and are clearly separated. From T5 to T8, stripes sharpen, becoming progressively narrower. Posterior stripes 5–7 can be seen to shift to the anterior. Such dynamic anterior shifts of posterior *eve* stripes are also observed in *D. melanogaster* [38].

### Extraembryonic tissues in *M. abdita*

Another aspect of *M. abdita* embryogenesis that has been carefully investigated is the formation and development of the extraembryonic tissues (the amnion and the serosa; [24]–[26]. One of the reasons for this is that it is one of the most divergent morphological traits involved in the early development of dipterans, and its evolution is closely linked to that of axis formation and early embryonic patterning [45]. While *D. melanogaster* and other schizophoran flies exhibit a much reduced dorsal amnioserosa, other dipteran species, such as *M. abdita*, have more fully developed and separated amniotic and serosal tissues [25], [46].

**Figure 9:**
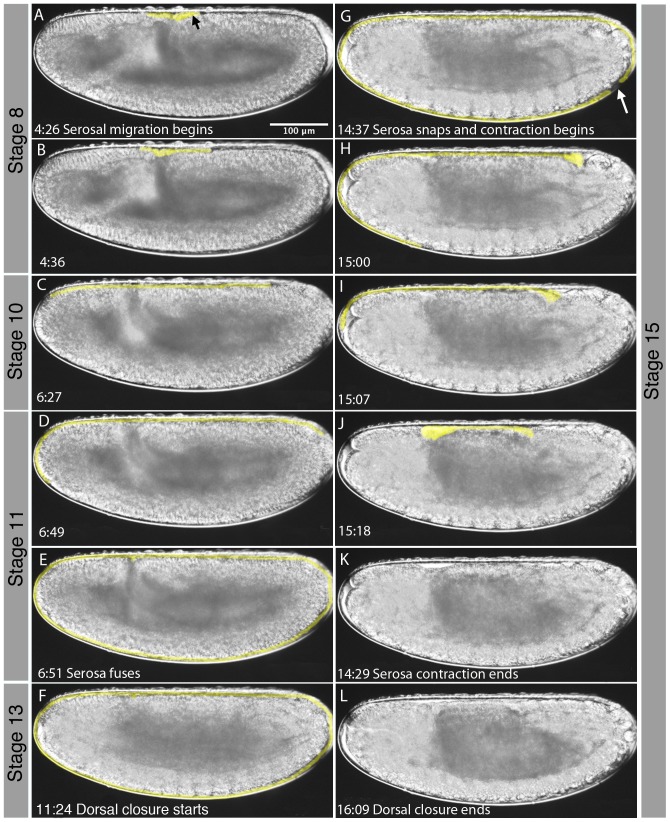
Extension and retraction of the serosa in *M. abdita*. Time is shown in hrs:min after egg laying (AEL) for each image. The serosa is highlighted in yellow. The black arrow in (A) indicates the amniosersal lip, the white arrow in (G) the position where the serosa ruptures. Corresponding embryonic stages (see Figures 2 and 3) are indicated on grey background. See text for details.

In what follows, we characterise morphological aspects of the formation and development of extraembryonic tissues in *M. abdita*. This process takes place between stage 8, towards the end of the rapid phase of germband extension, and stage 15, when the serosa breaks (see Figure 9). The first morphological sign of the presence of extraembryonic tissues is the formation of the amnioserosal lip at the posterior end of the extending germband around 4 hrs 10 min AEL (Figure 9A, black arrow). Serosal migration starts soon after. Although this is not detectable in our movies, the serosa must detach from the underlying head epithelium, before migrating to the front, rounding the anterior pole (Figure 9B–D). The serosa also extends posteriorly, and ultimately fuses in a ventro-posterior position (Figure 9C–E). This process takes around 2 hrs 9 min. During this time, the germband has reached its maximum extent. The amnion stays confined to the region of the dorsal opening of the embryo (see [25]).

The serosa covers the embryo through germband retraction and the early stages of dorsal closure and head involution (Figure 9F). Then it abruptly ruptures in a ventro-posterior position, 2 hrs 25 after the onset of dorsal closure, 1 hr 23 min after the onset of head involution and around 9 hrs after starting its migration (Figure 9F, white arrow). Rupturing of the serosa may be due to forces exerted by the progressing process of dorsal closure. As a consequence, serosal tissue is rapidly retracted towards the dorsal opening, taking only about 10 min to round the posterior (Figure 9G–H), and about 19 min to round the anterior pole (Figure 9G–I). Contraction ends 40 min after rupturing as dorsal closure continues (Figure 9J–L). Both amnion and serosal tissues are reabsorbed into the dorsal opening at this stage while dorsal closure takes another 2 hrs 20 min to complete. Due to limitations in DIC optics it is not clear whether the retracted extraembryonic tissues form a dorsal organ such as the one observed in *D. melanogaster*.

### Germ line development: formation of the pole cells

The germline of *D. melanogaster* differentiates from the somatic lineage early in development. This process can be observed at the morphological level as the formation of posterior pole buds at stage 3 [27]. The pole buds divide once during this stage, and once more at stage 4, before pinching off to form 12–14 pole cells. A second division in stage 4 results in 34–37 pole cells.

**Figure 10:**
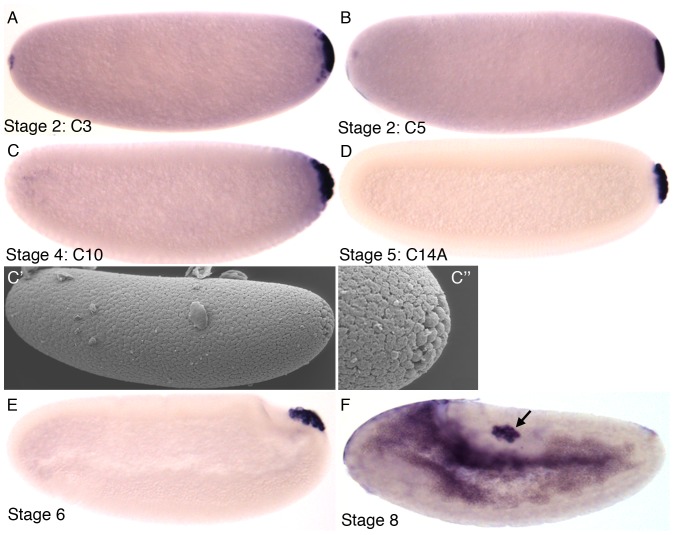
Pole cell formation in *M. abdita*. (A–D, E, F) Embryos stained with antibodies against Vasa protein at cleavage cycles C3, C5, C10, C14A (stages 1–5), as well as stage 6 and stage 8. Lateral views, anterior is to the left, dorsal is up. Arrow in (F) indicates the position of the pole cells after their inwards migration. (C') scanning electron micrograph (SEM) of C10 embryo; close-up in (C'') shows pole cells.

Germ cells are targeted in the process of making transgenic flies. Therefore, precise knowledge of their formation and location in *M. abdita* is likely to be of use when attempting transgenesis. We identified pole buds and pole cells by morphology, and via the highly conserved germline marker protein Vasa. We first detect Vasa protein at C3 (stage 2) at the posterior pole of the embryo, before the formation of the pole buds (Figure 10A). Posterior Vasa expression is maintained through C5, and becomes localised to the pole buds as they emerge during stage 3, and to the pole cells as they pinch off at stage 4 (Figure 10B–D). A scanning electron micrograph (SEM) of a C10 embryo shows the presence of bulges at the posterior of the embryo representing the pole cells (Figure 10C', and magnified inset C''). During gastrulation and germband extension, Vasa continues to mark the pole cells as they start their movement inside the embryo at stages 6 to 8 (Figure 10E–F). Both the expression of Vasa and the location and movement of the germ line are identical to *D. melanogaster* [47]–[49].

## Conclusion

In this paper, we provide a detailed and systematic characterisation of the life cycle and embryonic development of the scuttle fly *M. abdita*, while the accompanying study by Jiménez-Guri et al. [9] does the same for the moth midge *C. albipunctata*. These two papers provide a valuable resource and reference for the growing community of fly geneticists and evolutionary developmental biologists studying non-drosphilid dipteran species.

In particular, we propose an embryonic staging scheme (Figures 2 and 3), which is homologous to the one already established for *D. melanogaster* [27]. In addition, we investigate a number of developmental processes in more detail. We have determined the number of cleavage divisions before the onset of gastrulation (Figure 4), have measured the exact length of cleavage cycles (Figure 5), and establish morphological markers for the precise staging of embryos at the blastoderm stage (Figures 6 and 7). We illustrate the use of this staging scheme by describing the dynamics of *eve* expression at high temporal resolution (Figure 8). In addition, we describe the morphology and dynamics of extraembryonic tissue formation and retraction (Figure 9). Finally, we examined germ line development by describing the formation of pole buds and pole cells during early development (Figure 10).

Our study shows that embryogenesis and life cycle characteristics (such as the number of larval instars) are highly conserved across cyclorrhaphan flies. Despite the large evolutionary distance between them, embryogenesis of *M. abdita* and *D. melanogaster* are extremely similar, both with regard to timing and morphological characteristics. The most obvious difference in *M. abdita* compared to *D. melanogaster* consists of the formation and retraction of fully formed extraembryonic tissues (see also [24]–[26]).

## Materials and Methods

### Fly culture and embryo collection

*M. abdita* embryos were collected after 5–10 min laying time, and dechorionated as described in [50], [51]. To image the embryos we brushed the dechorionated embryos onto a microscopy slide and covered them with a drop of 10S voltalef oil ensuring that the embryos did not dry out. Live imaging typically started 10–20 min after egg laying.

### Life cycle imaging

Adult and larval stage images for Figure 1 were captured using a Leica EC3 camera mounted on a dissecting stereoscope. A light diffuser consisting of a cylinder of white paper was used to spread light from the light source evenly over a sample mounted on a glass needle. Multiple *z*-stacks of each sample were taken and in-focus regions patched together using Photoshop.

### Embryo imaging

Embryo images for Figures 4, 8, and 10 were taken using a Leica DM6000B upright compound microscope using a 10× objective. Pictures for DAPI counterstaining, *in situ* hybridisation, and antibody staining experiments were acquired and processed as described in [34].

### Time-lapse imaging

Slides were placed on a temperature-controlled platform at 25°C. Embryos were imaged with a Leica DM6000B upright compound microscope using 20×, 40×, or 63× objectives, and time intervals between image acquisitions ranging from every 10 s to every 1 min. Specifications of optics, magnification, camera, time interval, and embryo orientation for each time-lapse are provided in File S1. Movies were processed using ImageJ (http://rsbweb.nih.gov/ij).

### Nuclear staining

DAPI counterstains were performed as follows: fixed, methanol-dehydrated embryos [50], [51] were rehydrated into PBT and incubated with PBT/DAPI (0.3 µM DAPI) for 10 min. Embryos were washed 3× in PBT for 1 min, followed by longer washes of 3×10 min in PBT. Stained embryos were mounted and stored in 70% glycerol/PBS.

### *In Situ* Hybridisation

*In situ* hybridisation was carried out as described in [34]

### Antibody Staining

Immunostainings were performed using an antibody against Vasa protein (kindly provided by P. Lasko) at 1∶250 dilution. Fixed embryos, stored in methanol (see previous section), were rehydrated for 5 min in PBT/Methanol (1/1) and washed in PBT (2×1 min, 1×20 min) at room temperature (as were all subsequent stages unless indicated). Blocking was carried out with 2×30 min washes in PBT with Western Blocking Reagent (PBTB) (Roche). Incubation with primary antibody was in PBTB for 3 hrs. 3× PBT washes were performed followed by a final overnight wash in PBT at 4°C. Blocking for the secondary antibody was performed as described previously. Incubation with secondary antibody (goat anti-rabbit, 1∶3000; Jackson ImmunoResearch Laboratories, Inc.) was carried out in PBTB for 1 hr. Washes were 3× in PBT and 4×15 min in PBT. Pre-stain washes were 2×5 min in AP Buffer (100 mM NaCl, 50 mM MgCl_2_, 100 mM Tris PH 9.5, 0.1% Tween). Staining was performed in AP Buffer with 1 µl/ml NBT and BCIP (Roche). After staining, embryos were washed in PBT, followed by DAPI staining and mounting as described above.

### Scanning Electron Microscopy

Scanning electron micrographs were taken with a Zeiss DSM 940A scanning electron microscope at the Unitat de Microscopia Electronica (Campus Casanova) of the University of Barcelona. Samples were processed as follows: samples were fixed using 2.5% glutaraldehyde in 0.1 M cacodylate buffer overnight at 4°C, followed by 3×10 min washes at 4°C in 0.1 M cacodylate buffer. Post-fixation was carried out in 1% osmium tetroxide in 0.1 M cacodylate buffer for 2 h at 4°C followed by 3×10 min washes at 4°C in milliQ water. Embryos were put through an ethanol dilution series of 25, 50 and 70%, each for 10 min at 4°C, then 3×10 min additional washes in 90, 96 and 100% ethanol at 4°C. Embryos were critical-point-dried using a VGMicrotech CPD 7501 system, and gold coating was carried out using a Fisons Instrument FC510 Sputtering System.

## Supporting Information

File S1 **Timing of developmental events from individual time-lapse movies in** ***M. abdita.*** Stages and developmental events are shown in columns A and B. Time-lapse (TL) movie IDs are listed along the top, along with averages of timing of events across embryos/movies in minutes, and hours:minutes (hh:mm). Also listed are the number of embryos *n* underlying the calculation of average times for each event, stage duration (in min and in hh:mm) and standard deviation (STDEV, in min) for each event. % of developmetal time is also shown for each stage. Row 2 indicates the time adjustment made to each event to cancel out variations in starting time; 10 min were added for each unrecorded cleavage cycle (i.e. in TL29, C4 starts at 8 min 30 s in the raw data, therefore the start of the movie is C3 + 2 min 30 s; by adding 2.5 min we arrive at the start of C3, by adding 10 min at the start of C2, and by adding another 10 min at the start of C1; hence, to normalise this movie, we add 2.5+10+10 = 22.5 min). Rows 3–5 detail the optics (20×, 40× or 64×), camera (Leica DFC420 or DFC360FX), and the embryo view (full, dorsal, ventral or posterior). Row 6 gives the time interval between capturing successive images. Alternating white and grey rows mark stages.
(XLS)

File S2 **Nuclei number and density counts for pre-gastrulation embryos of** ***M. abdita.*** Nuclear counts (orange table) are shown below expected numbers for each cleavage cycle (C1 to C14). Nuclear density counts (red table) are shown for cleavage cycles C12–C14. Nuclear density was assessed by scaling each embryo to 600×250 mm and counting the number of full nuclei falling into a 45×4 mm square placed in the middle of the embryo.
(XLS)

Movie S1 **Time-lapse movie covering the entire embryonic development of** ***M. abdita*** **.** Time-lapse movie of a *M. abdita* embryo taken using a 20× objective and DIC optics under 10S voltalef oil. Lateral view: anterior is to the left, dorsal is up. This movie corresponds to TL29 in File S1.
(MOV)

Movie S2 **Time-lapse movie covering the blastoderm stage of** ***M. abdita*** **.** Time-lapse movie of a *M. abdita* embryo taken using a 20× objective and DIC optics under 10S voltalef oil. Lateral view: anterior is to the left, dorsal is up. This movie corresponds to TL26 in File S1.
(MOV)
